# World Health Organization (WHO) International Classification of Functioning, Disability and Health (ICF) Core Set Development for Interstitial Lung Disease

**DOI:** 10.3389/fphar.2022.979788

**Published:** 2022-10-14

**Authors:** Lesley Ann Saketkoo, Reuben Escorpizo, Janos Varga, Kevin John Keen, Kim Fligelstone, Surinder S. Birring, Helene Alexanderson, Henrik Pettersson, Humza Ahmad Chaudhry, Janet L. Poole, Malin Regardt, Daphne LeSage, Catherine Sarver, Joseph Lanario, Elisabetta Renzoni, Mary Beth Scholand, Matthew R. Lammi, Otylia Kowal-Bielecka, Oliver Distler, Tracy Frech, Lee Shapiro, Cecilia Varju, Elizabeth R. Volkmann, Elana J. Bernstein, Marjolein Drent, Ogugua Ndili Obi, Karen C. Patterson, Anne-Marie Russell, The Global Fellowship on Rehabilitation and Exercise in Systemic Sclerosis (G-FoRSS)

**Affiliations:** ^1^ New Orleans Scleroderma and Sarcoidosis Patient Care and Research Center, New Orleans, LA, United States; ^2^ University Medical Center—Comprehensive Pulmonary Hypertension Center & Interstitial Lung Disease Clinic Programs, New Orleans, LA, United States; ^3^ Tulane University School of Medicine, New Orleans, LA, United States; ^4^ Louisiana State University Health Sciences Center, Division of Pulmonary Medicine—New Orleans, New Orleans, LA, United States; ^5^ Department of Rehabilitation and Movement Science, The University of Vermont, Burlington, VT, United States; ^6^ Swiss Paraplegic Research, Nottwil, Switzerland; ^7^ Department of Pulmonology, Semmelweis University, Budapest, Hungary; ^8^ Department of Mathematics and Statistics and Health Research Institute, University of Northern British Columbia, Prince George, BC, Canada; ^9^ Department of Medicine, University of British Columbia & Centre for Heart Lung Innovation, Providence Research, Vancouver, BC, Canada; ^10^ Patient Research Partner Scleroderma & Raynaud Society, UK (SRUK) and Federation of European Scleroderma Associations, London, United Kingdom; ^11^ Royal Free Hospital Scleroderma Unit, London, United Kingdom; ^12^ Division of Asthma, Allergy and Lung Biology, King’s College London, London, United Kingdom; ^13^ Women’s Health and Allied Health Professionals, Medical Unit Occupational Therapy and Physiotherapy, Karolinska University Hospital, Stockholm, Sweden; ^14^ Department of Medicin, Division of Rheumatology, Karolinska Institutet, Stockholm, Sweden; ^15^ Occupational Therapy Graduate Program, University of New Mexico, Albuquerque, NM, United States; ^16^ Patient Research Partner, New Orleans, LA, United States; ^17^ Patient Research Partner, Baltimore, MD, United States; ^18^ Research Fellow in Respiratory Health—Exeter Respiratory Institute Royal Devon University Hospitals NHS Foundation Trust, Exeter, United Kingdom; ^19^ Royal Brompton Hospital, National Heart and Lung Institute, London, United Kingdom; ^20^ Pulmonary Medicine, University of Utah, Salt Lake City, UT, United States; ^21^ University of Bialystok, Bialystok, Poland; ^22^ Division of Rheumatology, University Hospital Zurich, Zurich, Switzerland; ^23^ Division of Rheumatology Vanderbilt University School of Medicine, Nashville, TN, United States; ^24^ Pulmonary Medicine, University of Utah, Salt Lake City, UT, United States; ^25^ Division of Rheumatology, Albany Medical Center, Albany, NY, United States; ^26^ Steffens Scleroderma Foundation, Albany, NY, United States; ^27^ Department of Rheumatology and Immunology, Medical School, University of Pécs, Pecs, Hungary; ^28^ Department of Medicine, David Geffen School of Medicine, UCLA Scleroderma Program and UCLA CTD-ILD Program, Division of Rheumatology, University of California, Los Angeles, Los Angeles, CA, United States; ^29^ Department of Medicine, Columbia University/New York-Presbyterian Scleroderma Program, Division of Rheumatology, Columbia University College of Physician2s and Surgeons, New York, NY, United States; ^30^ Department of Pulmonology, Interstitial Lung Diseases (ILD) Center of Excellence, St. Antonius Hospital, Nieuwegein, Netherlands; ^31^ Department of Pharmacology and Toxicology, Faculty of Health and Life Sciences, Maastricht University, Nieuwegein, Netherlands; ^32^ Department of Internal Medicine, Division of Pulmonary, Critical Care and Sleep Medicine, Brody School of Medicine, East Carolina University, Greenville, NC, United States; ^33^ Department of Clinical & Experimental Medicine, Brighton & Sussex Medical School, Falmer, United Kingdom; ^34^ Division Pulmonary, Allergy, and Critical Care Medicine, Perelman School of Medicine, University of Pennsylvania, Philadelphia, PA, United States; ^35^ Respiratory Institute to Exeter Respiratory Innovation Center, University of Exeter, Exeter, United Kingdom; ^36^ Respiratory Medicine, Royal Devon University Healthcare NHS Foundation Trust, London, United Kingdom

**Keywords:** fibrosis, ICD-11, patient-reported outcomes, connective tissue, cough, CTD-ILD

## Abstract

**Background:** The World Health Organization (WHO) introduced the International Classification of Functioning, Disability, and Health (ICF) as a scientific method of disability data collection comprised of >1,200 categories describing the spectrum of impairment types (functional, symptoms-based and anatomical) under the bio-psycho-social model with consideration of *environmental* and *personal factors* (pf). ICF Core Sets and ICF Checklists are streamlined disease-specific resources for clinical use, service provision, and for use in health economics and health policy. ICF can disclose strengths and weaknesses across multiple patient-reported outcome measures (PROMs) and help consolidate best-fitting question-items from multiple PROMs. Interstitial lung diseases (ILDs), are generally progressive, with restrictive physiology sometimes occurring in the context of multi-organ autoimmunity/inflammatory conditions such as connective tissue diseases (CTDs). In spite of significant associated morbidity and potential disability, ILD has yet to be linked to the ICF.

**Methods:** Each instrument and their question-items within the consensus-recommended core sets for clinical trials in ILD were deconstructed to single concept units, and then linked per updated ICF linkage rules. Inter-linker agreement was established. Three additional subsequently validated measures were also included.

**Results:** One-hundred-eleven ICF categories were identified for ten PROMs and three traditional objective measures that were amenable to ICF linkage. The proportion of agreement ranged from 0.79 (95% CI: 0.62, 0.91) to 0.93 (0.76, 0.99) with the overall proportion of inter-linker agreement being very high 0.86 (0.82, 0.89) for the initial instruments, with 94–100% for the three additional PROMs. Thirty-four new ‘Personal Factors’ emerged to capture disease-specific qualities not elsewhere described in ICF, e.g. ‘pf_embarrassed by cough’ or ‘pf_panic/afraid when can’t get a breath’.

**Conclusion:** This first known effort in ICF linkage of ILD has provided important revelations on the current utility of the ICF in lung disease. Results have indicated areas for meaningful assessment of ICF descriptors for lung impairment. The mapping across PROMs provides insight into possibilities of developing more streamline and precise instrumentation. Finally, familiarity with the ICF in ILD may enable clinicians to experience a smoother transition with the imminent harmonization of ICD and ICF, ICD-11.

## Introduction

Interstitial lung diseases (ILD) are a heterogeneous group of predominately restrictive lung diseases imparting significant morbidity and mortality (Travis et al., 2013; Wijsenbeek and Cottin, 2020). Idiopathic pulmonary fibrosis (IPF) is a progressive pauci-immune fibrotic process predominantly involving the lungs and with no known cure (Spagnolo et al., 2018). Connective tissue diseases (CTD) such as systemic sclerosis, inflammatory myopathy, Sjogren’s syndrome and rheumatoid arthritis (RA) affect the lungs in the setting of other systemic/multi-organ autoimmunity with radiographic and pathological patterns varying in degrees of inflammatory and/or fibrotic progression (Wijsenbeek and Cottin, 2020; Ruaro et al., 2021). Through iterative medical expert and patient consensus methodology (Saketkoo et al., 2014a; Saketkoo et al., 2014b), recent efforts identified a minimum set of instruments for inclusion in clinical trials and longitudinal observational studies in IPF and CTD-ILD, respectively.

**FIGURE 1:**
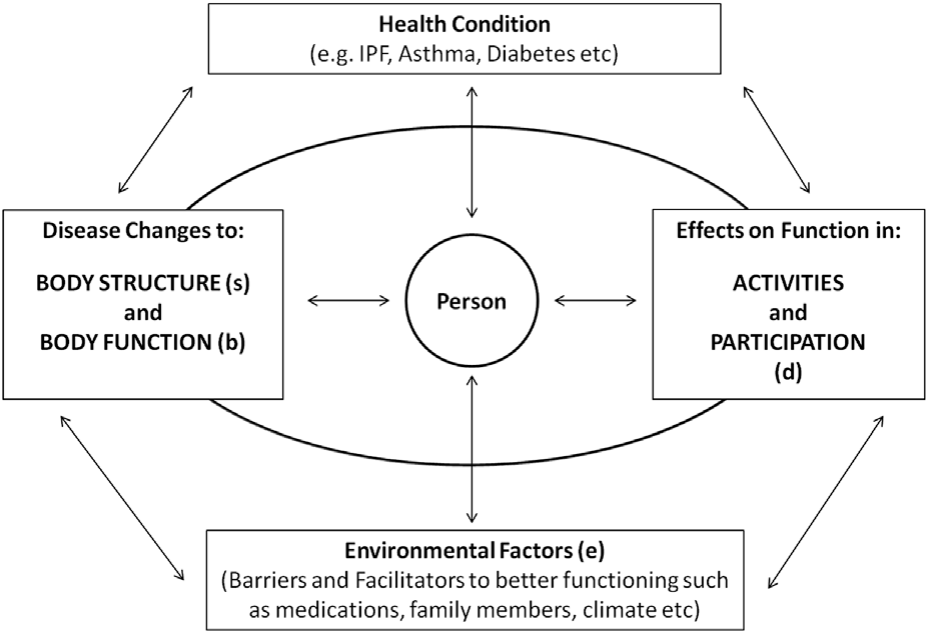
Interactions between domains of ICF in relation to the health condition. *Courtesy of LA Saketkoo, with permission, rights reserved.*

The World Health Organization’s (WHO) International Classification of Functioning, Disability and Health (ICF) is an alphanumeric classification system integrating the bio-psychosocial model of health and was officially adopted by the World Health Assembly in 2001 (Figure 1 and online supplement.).

As with the WHO’s other classification system, the International Classification of Diseases (ICD), a system originally developed to quantify burden of specific health conditions (diagnoses), the ICF can be used on global, national, regional, local, and institutional levels. The ICF was originally devised as a standardized means to scientifically assess the global burden of disability and chronic disease (World Health Organization, 2001; World Health Organization Website, 2015), thus providing guidance for reimbursement, infrastructural support, allotment of research and service funding, and, importantly, policymaking. Through a hierarchical mechanism of over 1,200 “categories” (or codes) that depict functioning and disability (World Health Organization International Classification of Functioning Disability and Health, 2001), the ICF attempts to describe the spectrum of impairment type (symptomatic and anatomical) and to quantify the burden of disability of a population, as well as capturing “environmental factors” that either improve function and mitigate disability (such as ramps, assistive technology, medications, supportive family, etc), or that worsen impairment (staired entries, lack of accessible transport, unaffordable assistive aids, etc.).

Beyond epidemiologic and health economics use, the ICF has emerged to be highly versatile and multi-purpose in its utility including clinical assessment of specified health conditions, It initially focused on rehabilitative care (e.g. traumatic brain injury, stroke etc.) and subsequently expanded to other chronic health conditions (e.g. RA, diabetes, or obesity). Clinical applications of the ICF gave rise to the development of ICF Core Sets (collections of ICF categories relevant to a disease) along with ICF Checklists (clinical forms with selected categories from the ICF Core Set), abbreviated collections of ICF categories/codes for specific health conditions that facilitate assessment of symptom burden, impairment, treatment response, side effects and service needs.

ICF Core Sets and Checklists can be administered online or on paper, and are patient or clinician reported. ICF is multi-purpose and can be used along with the Patient Specific Functional Scale St (PSFS) (Stratford et al., 1995; Fairbairn et al., 2012; Patient specific functional scale, 2021), MACTAR (Alemo Munters et al., 2014) or Canadian Occupational Performance Measure (COPM) (Stamm et al., 2004) to help identify and prioritize unique preferences in functional achievements. Similarly, ICF can help disclose strengths and weaknesses across multiple patient-reported outcome measures (PROMs) and consolidate best-fitting question items.

The ICD and ICF classification systems complement each other, and the current WHO intention is to harmonize these two classification systems for ICD-11 (Escorpizo et al., 2013). The Center for Medicare and Medicaid Services (CMS) in the United States (Iezzoni and Greenberg, 2003; Health and Human Services/Centers for Medicare and Medicaid Services, 2000; SocialSecurity Administration, 2013; Escorpizo and Stucki, 2013) has adopted the 11th revision of the ICD, which will integrate the ICD-10 and ICF classifications by simultaneously conveying the diagnosis with the type and degree of impairment (Kohler et al., 2012; Escorpizo et al., 2013; Selb et al., 2015a; Selb et al., 2015b). Our goal in linking ILD to the ICF is the development of disease-specific ICF Core Sets to aid pulmonary-focused clinicians in this transition (Saketkoo et al., 2012a; Selb et al., 2015a). The results provide significant utility beyond our original goals, including ICF language enhancements and mapped variations of current PROMs that may inform improved patient-reported instrumentation.

## Methods

The goals of the study are to produce a feasible approach to ICF for clinicians and researchers working in interstitial lung disease, and to foster confidence and familiarity with the ICF during the ICD-11 transition (Escorpizo et al., 2013). Additionally, the study interrogates for needed modifications and updates to advance the current ICF content in pulmonary disease.

### Structure of the ICF classification

The ICF Classification consists of two over-arching parts with independent components:

1. *Functioning and Disability,* the predominant operational part of the classification consists of:
a) *“Body Structure”* (“**s**” terms) are *abnormalities of anatomical structure*, such as that of lung parenchyma (s4301)
b) *“Body Function”* (“**b**” terms) are physiologic functions including *symptomatic experience* of physical, mental and emotional functions e.g. energy/fatigue (b1300), dyspnea (b460), cough (b450) or chest pain (b28011)
c) *“Activities and Participation”* (“**d**” terms) are defined under “activities” e.g. lifting (d4300), bathing (d510), cooking (d630), or moving between locations (d460); and under “participation” such as life situations with work (d850) or family (d760).

2. *Contextual Factors* are divided into:

a) external or *“Environmental Factors”* (“**e**” terms) that either positively (e.g., personal assistive devices, e1151) or negatively (e.g., inaccessible transportation, e120) impact functioning
b) internal or *“Personal Factors”*, a developing ICF area, are individualized factors that potentially influence disability e.g., gender, age, coping styles, behavior and psychological characteristics.

Categories are tiered into “levels” of specificity (e.g., b5 digestive system, b510 ingestion, b5105 swallowing, b51052 esophageal swallowing).

### Linking the CTD-ILD and IPF consensus instruments to the ICF

A diverse expert team of clinicians, patients and researchers provided responsive feedback regarding instrument selection, analysis and interpretation. These consensus measures included seven vetted PROMS and four “objective” measures considered for use in clinical trials for IPF and for CTD-ILD (Saketkoo et al., 2014b) (Table 1) as well as three other subsequently validated PROMs (Patel et al., 2012; Russell et al., 2017; Russell, 2018; Swigris et al., 2020) that were included because of anticipated high utilization. Each of these measures was attempted to be linked to ICF categories (Table 2) by two independent investigators (RE,LAS) according to updated ICF linking rules (Cieza et al., 2005). (As the *Short Form Health Survey (SF-36)* was previously linked by ICF scholars, these linkages were used.) To accomplish the linking, each of the measures were deconstructed to its most basic single-concept units, which required PROM question-items to each be deconstructed. For example, an item querying “mowing lawn makes me breathless” is comprised of two discrete concepts linked individually to ICF, “mowing lawn” and “breathless”. Each concept-item, however, may evoke more than one linkage, such as “mowing lawn” suggests “pushing an object” and “caring for home”. If any item analysis identified discordance, these were resolved between the linkers. This was done by each linker stating their position of support for the items they had chosen, then each stating if they had any positions of disagreement for the discordant items chosen by the other. This was then followed by discussion of each item to keep or dismiss. Irresolvable disagreement between linkers on an item would be decided by at least one person also trained in the ICF (AMR,HA,HP,OD).

**TABLE 1:** Consensus on minimal core sets of instruments and measures for IPF and CTD-ILD trials (Saketkoo et al., 2014b)

Domain	Instruments/Measures	CTD-ILD	IPF
Dyspnea (Breathlessness)	Medical Research Council Chronic Dyspnea Scale	+	+
	Dyspnea 12	+	+
	UCSD-Shortness of Breath Questionnaire	-	+
Cough	Leicester Cough Questionnaire	+	+
HRQoL	Medical Outcomes Study Short Form-36	+	+
	St. Georges Respiratory Questionnaire	+	+
	VAS Patient Global Assessment of Disease Activity (VAS-PtGA)	+	−
Lung Imaging	Overall Extent of ILD on HRCT	+	+
Lung Physiology	Forced Vital Capacity (FVC)	+	+
	Diffusion Capacity of Lung (DLCO)	+	+
Survival	All Cause Mortality	+	+
**Additional PROMs Validated After Consensus Project with Anticipated High Utility**			
	Pulmonary Fibrosis PROM (PF-PROM)^	^	^
	King’s Brief ILD Questionnaire (K-BILD)^	^	^
	Living with IPF Questionnaire (LIPF)^	^	^

“+” signifies “*part”* and “−”signifies “*not part*” of minimal core set of consensus instruments for IPF, or CTD-ILD, clinical trials, ^ signifies validated subsequent to consensus. (Travis et al., 2013).

**TABLE 2:** Distribution of ICF categories and instrument occurrence per domain with example linkages. (*Courtesy of LA Saketkoo, with permission, rights reserved*)

ICF domain	Description	Instruments linked	No. of ICF categories linked	Examples from CTD-ILD and IPF core sets
Body Structure	Relates to involvement of anatomical structures	HRCT	1	s4301, Structure of lungs
Body Function	Relates to physical, mental and emotional functions including symptoms	D-12, DLCO, FVC, LCQ, MRC-DS, PF/IPF-PROM, K-BILD, LIPF, SF-36, SGRQ, UCSD-SOBQ	28	b1300, Energy level
				b134, Sleep functions
				b4402, Depth of respiration
				b455, Exercise tolerance
				b28011, Pain in chest
Activities and Participation	Execution of task or action; involvement in daily and overall life situation	D-12, LCQ, MRC-DS, PF/IPF-PROM, K-BILD, LIPF, SF-36, SGRQ, UCSD-SOBQ	71	d330, Speaking
				d430, Lifting, carrying objects, d4600, Moving around house
				d510, Washing oneself
				d8451, Maintaining a job
Environmental Factors	**Positive** (e.g. family, medications, assistive devices, oxygen, lifts) or **Negative** (e.g. stairs, lack of income, cold climate, distance from services) influences on performance	LCQ, K-BILD, LIPF, SGRQ	11	Products and technology for personal use in daily living e115_oxygen supplementation, Financial assets e1650, Tangible assets e1651, e260, Air quality e460, Societal attitudes
				e2100, Land forms, such as mountains, hills, valleys and plains
Total			**111**	

Pre-resolution inter-linker agreement was analyzed (KJK) for each instrument with the estimates of the proportion of agreement and confidence intervals according to the exact binomial test using release 3.1.0 of the R statistical software package (Core Team, 2014).

## Results

One-hundred and eleven ICF categories were identified under the four ICF components (“*Body Structure”*, “*Body Function”*, “*Activities and Participation”*, and “*Environmental Factors”*) for nine patient-reported questionnaires and three traditional objective measures (Table 2).

### Linking agreement

Agreement between linkers was high (Table 3). The pre-resolution proportion of agreement ranged from 0.79 (95% CI: 0.62, 0.91) to 0.93 (0.76, 0.99) for the five-remaining consensus PROMs (Saketkoo et al., 2014b) (as no linking occurred for *Visual Analogue Scale-Patient Global Assessment of Disease Activity* (*VAS-PtGA*) and *SF-36*) with the overall proportion of inter-linker agreement 0.86 (0.82, 0.89). There was 100% agreement between the linkers for pulmonary function measures of *forced vital capacity* (*FVC*) and *diffusion capacity* (*DLCO*), and the *Overall Extent of ILD on HRCT*. There was 94–100% linking agreement for the three additional PROMs (*PF/IPF-PROM*, *King’s Brief Interstitial Lung Disease Questionnaire* (*K*-*BILD*), and *Living with IPF* (*LIPF*) *Questionnaire*). Initial linking discrepancies were resolved to 100% between linkers without need for arbitration.

**TABLE 3:** Instruments from the previously published CTD-ILD and IPF minimum core sets for clinical trials with instrument comparison and inter-reviewer agreement. (*Courtesy of LA Saketkoo, with permission, rights reserved*)

Consensus instruments for CTD-ILD and IPF (Saketkoo et al., 2014b)	Number of concept-items linked	Number of categories identified	Agreement (%)	Agreement 95% confidence interval
Medical Research Council (MRC) Dyspnea Scale	27	34	79%	(62, 91)
Dyspnea 12 (D-12)	25	27	93%	(76, 99)
University of California San Diego –Shortness of Breath Questionnaire (UCSD-SBQ)	68	83	82%	(72, 90)
Leicester Cough Questionnaire	44	56	79%	(66, 88)
St Georges Respiratory Questionnaire	126	138	91%	(85, 95)
Medical Outcomes Study Short Form 36 (SF-36)		26^a^	Previously linked version	
Visual Analogue Scale-Patient Global Disease Activity	Not defined by ICF (too broad)			
***Overall Initial Agreement on Consensus Questionnaires***	**290**	**338 (+26^a^)**	**86%**	**(82, 89)**
**Traditional Measures**				
Forced Vital Capacity on Spirometry	2	2^a^	100%	N/A
Diffusion Capacity of the Lung for Carbon Monoxide (DLCO)	1	1^a^	100%	N/A
Overall Extent of ILD on High Resolution CT (HRCT)	1	1^a^	100%	N/A
All Cause Mortality	Not defined by ICF (too broad)			
**Linker Agreement of Additional PROM Instruments**				
PF/IPF-PROM	—	—	100%	Not done
King’s Brief Interstitial Lung Disease Questionnaire (K-BILD)	—	—	100%	Not done
Living with IPF (LIPF) Questionnaire	—	—	94.3%	Not done

a Linked to an objective instrument and not from a questionnaire.

### Linkages

Of the combined total 111 linkages identified, 28 fell under “*Body Function”*, one under “*Body Structure*” (lung), 71 under “*Activities and Participation”* and 11 under “*Environmental Factors*”. *All-Cause Mortality* and the *VAS-PtGA* were not definable in ICF terms. *Extent of ILD on HRCT* was the only measure demonstrating linkage under “*Body Structure”* representing a single category, *s4301*, *“Structure of Lungs”*.

The ICF contained no direct and specific linkages for the pervasive ILD symptoms of *breathlessness* and *cough*. To address this, many new linkages created to temporarily accommodate the concepts held in the PROMs. However, we propose these enduring ICF additions:

• two new ICF categories under “*additional respiratory functions*”: “*cough*” *(b4501)* and “*phlegm production*” *(b4502)*,
• one new ICF category under “*respiratory functions*”: “*respiratory flow* including airflow interrupted by inspiratory cough”(*b4403*), and
• three under “*sensations associated with cardiovascular and respiratory functions*”: “*sensation of breathlessness*” *(b4600),* “*sensation of air hunger*” *(b4601),* “*wheezing*” *(b4602).*

Regarding concepts of high ILD relevance (Swigris et al., 2005; Saketkoo et al., 2014a; Saketkoo et al., 2014b) cited by people living with ILD, there appeared to variation of frequency across PROMs.

*Exercise tolerance* (*b455*), as did *respiratory symptoms* (*b440-b460*) demonstrated linkage in all PROMS except *SF-36*. Depth of respiration (*b4402*) was queried in two PROMs, the *Dyspnea-12* (*D-12*) and *LIPF.* “*Coughing”* in any form was noted in only three PROMs: *LCQ*, *SGRQ*, and *LIPF*. *“Cough with deep inspiration”* and “*coughing with over-exertion” were queried* in *LIPF* and *“bouts of coughing”* in *LCQ*. Linkages relating to voice quality (*b3101*), speaking (*d330*) and conversation (*d350,d3600*) were represented by the *LCQ* and *LIPF*.

Of nine PROMs, *Energy level, (b1300)* and *fatiguability (b4552)* was queried in six of the nine included PROMs (*D-12*, *LCQ, SF-36, SGRQ, PF/IPF-PROM, LIPF*). While *sleep (b134)* was queried in three PROMs (*LCQ, SGRQ, LIPF*). Ability to “*carry out daily routine” (d230)* demonstrated linkage in five PROMs (*LCQ, SF-36, PF/IPF-PROM, K-BILD, LIPF*); but only the LIPF queried complexity of task performance and did so in multiple dimensions (*d210, d2100, d2102, d220, d2202*) as well as self-pacing (*d2309_pace self*). While the *University of California San Diego-Shortness of Breath Questionnaire (UCSD-SBQ)*, accounted for unique linkages (Table 4, 5) all of which occurred under “*Activities and Participation*” and related to ability to sit, stand, to perform domestic care and move around outside the house; while both the *UCSD-SBQ* and the *LIPF* demonstrated linking to more highly detailed levels of self-care activities such dental care, washing and grooming.

**TABLE 4:** Categories according to instrument (*Courtesy of LA Saketkoo, with permission, rights reserved*)

WHO ICF category	WHO ICF descriptor	MRC	D-12	UCSD-SBQ^	LCQ	SF-36	SGRQ	PF/IPF-PROM	K-BILD	LIPF	HRCT	FVC	DLCO
**Body Structure**													
s4301	Structure of lungs	—	—	—	—	—	—	—	—	—	HRCT	—	—
**Body Functions**													
b1263	Psychic stability	—	—	—	—	SF-36	—	—	—	—	—	—	—
b1300^a^	Energy level	—	—	—	LCQ	SF-36	—	PF/IPF-PROM	—	LIPF	—	—	—
b134	Sleep functions	—	—	—	LCQ		SGRQ	—	—	LIPF	—	—	—
b152	Emotional functions	—	—	—		SF-36	—	—	—		—	—	—
b280	Sensation of pain	—	—	—	—	—	SGRQ	—	—	—	—	—	—
b28011	Pain in chest	—	D-12	—	LCQ	—	—	—	—	—	—	—	—
b28012	Pain in stomach or abdomen	—	—	—	LCQ	—	—	—	—	—	—	—	—
b3101	Quality of voice	—	—	—	LCQ	—	—	—	—	—	—	—	—
b440^a^	Respiration functions: Functions of inhalation, gas exchange, and exhalation	—	—	—	—	—	—	—	K-BILD	—	—	FVC	DLCO
b4402	Depth of respiration	—	D-12	—	—	—	—	—	—	LIPF	—	—	—
b4408_sputum_phlegem production	Respiration Functions Other specified	—	—	—	LCQ	—	SGRQ	—	—	—	—	—	—
b4408_cough with deep inspiration	—	—	—	—	—	—	—	—	—	LIPF	—	—	—
b450^a^	Additional respiratory functions: Additional functions related to breathing, such as coughing, sneezing and yawning	MRC	—	—	LCQ	—	SGRQ	PF/IPF-PROM	—	LIPF	—	FVC	—
b450_cough/ing^a^	Additional respiratory functions	—	—	—	LCQ	—	SGRQ	—	—	LIPF	—	—	—
b455^a^	Exercise tolerance functions	MRC	D-12	UCSD-SBQ^	LCQ		SGRQ	PF/IPF-PROM	K-BILD	LIPF	—	—	—
b455_physical exertion	—	—	—	—	—	—	—	—	—	LIPF	—	—	—
b455_stamina	—	—	—	—	—	—	—	—	—	LIPF	—	—	—
b4550	General physical endurance	—	—	—	LCQ	—	—	—	—	—	—	—	—
b4552^a^	Fatigability	—	D-12	—	LCQ	—	SGRQ	PF/IPF-PROM	—	—	—	—	—
b460^a^	Sensations associated with cardiovascular and respiratory functions: such as skipped heart beat, palpitation and shortness of breath	MRC	D-12	UCSD-SBQ^	—	—	SGRQ	Pf/IPF-PROM	K-BILD	LIPF	—	—	—
b460_air hunger/gasp	Ibid	—	—	—	—	—	—	—	K-BILD	—	—	—	—
b460_chest tightness	Ibid	—	—	—	—	—	—	—	K-BILD	—	—	—	—
b460_cough/ing^a^	Ibid	—	—	—	LCQ		SGRQ	—	—	LIPF	—	—	—
b460_wheeze/whistling sound	ibid	—	—	—	—	—	—	—	K-BILD	—	—	—	—
b469_at rest	Additional functions and sensations of the cardiovascular and respiratory systems, other specified and unspecified	—	—	—	—	—	—	—	—	LIPF	—	—	—
b469_bouts of coughing	ibid	—	—	—	LCQ	—	—	—	—	—	—	—	—
b469_cough/ing	Ibid	—	—	—	LCQ	—	—	—	—	LIPF	—	—	—
b469_cough/ing with deep inspiration	Ibid	—	—	—		—	—	—	—	LIPF	—	—	—
b469cough/ing with over-exertion	Ibid	—	—	—	—	—	—	—	—	LIPF	—	—	—
**Activity and Participation**													
d2	General tasks and demands	—	—	—	—	—	—	—	K-BILD	LIPF	—	—	—
d210	Undertaking a single task	—	—	—	—	—	—	—	—	LIPF	—	—	—
d2100	Undertaking a simple task	—	—	—	—	—	—	—	—	LIPF	—	—	—
d2102	Undertaking a single task independently	—	—	—	—	—	—	—	—	LIPF	—	—	—
d220	Undertaking multiple tasks	—	—	—	—	—	—	—	—	LIPF	—	—	—
d2202	Undertaking multiple tasks independently	—	—	—	—	—	—	—	—	LIPF	—	—	—
d230^a^	Carrying out daily routine	—	—	—	LCQ	SF-36	—	PF/IPF-PROM	K-BILD	LIPF	—	—	—
d299	General tasks and demands, unspecified	—	—	—	—	—	—	—	—	LIPF	—	—	—
d3	Communication	—	—	—	—	—	—	—	K-BILD	LIPF	—	—	—
d330	Speaking	—	—	—	LCQ	—	—	—	—	—	—	—	—
d350	Conversation	—	—	—	LCQ	—	—	—	—	LIPF	—	—	—
d3600	Using telecommunication devices	—	—	—	LCQ	—	—	—	—	—	—	—	—
d4	**Mobility**	—	—	—	—	—	—	—	—	LIPF	—	—	—
d4102	Kneeling	—	—	—	—	SF-36	—	—	—	—	—	—	—
d4103	Sitting	—	—	UCSD-SBQ^	—		—	—	—	—	—	—	—
d4104	Standing	—	—	UCSD-SBQ^	—		—	—	—	—	—	—	—
d4105^a^	Bending	—	—	UCSD-SBQ^	—	SF-36	SGRQ	—	—	—	—	—	—
d430^a^	Lifting and carrying objects	—	—	—	—	SF-36	SGRQ	—	K-BILD	LIPF	—	—	—
d4300	Lifting	—	—	—	—	SF-36	—	—	—	LIPF	—	—	—
d4301	Carrying in the hands	—	—	—	—		—	—	—	LIPF	—	—	—
d435	Moving objects with lower extremities	—	—	—	—	SF-36	—	—	—	—	—	—	—
d440	Fine hand use	—	—	—	—	SF-36	—	—	—	—	—	—	—
d445	Hand and arm use	—	—	—	—	SF-36	—	—	—	—	—	—	—
d4451	Pushing	—	—	UCSD-SBQ^	—	SF-36	—	—	—	—	—	—	—
d449	Carrying, moving and handling objects, other specified and unspecified	—	—	—	—	SF-36	—	—	—	—	—	—	—
d450^a^	Walking	MRC	—	UCSD-SBQ^	—	SF-36	SGRQ	PF/IPF-PROM	—	LIPF	—	—	—
d4500^a^	Walking short distances	MRC	—	—	—	SF-36	—	—	—	LIPF	—	—	—
d4501	Walking long distances	MRC	—	—	—	SF-36	—	—	—	—	—	—	—
d4502^a^	Walking on different surfaces	MRC	—	UCSD-SBQ^	—	—	SGRQ		K-BILD	—	—	—	—
d4508_walknig for periods^a^	Walking, other specified	MRC	—	UCSD-SBQ^	—	—	—	PF/IPF-PROM	—	—	—	—	—
d4508_walking pace	Walking, other specified	MRC	—	—	—	—	—	PF/IPF-PROM	—	—	—	—	—
d455	Moving around	—	—	—	—	—	SGRQ	—	—	—	—	—	—
d4551^a^	Climbing	—	—	UCSD-SBQ^	—	SF-36	SGRQ	—	K-BILD	LIPF	—	—	—
d4552	Running	—	—	—	—	SF-36	SGRQ	—	—	—	—	—	—
d4554	Swimming	—	—	—	—	—	SGRQ	—	—	—	—	—	—
d460	Moving around in different locations	—	—	—	—	—	—	—	—	LIPF	—	—	—
d4600	Moving around within the home	—	—	—	—	—	SGRQ	—	—	LIPF	—	—	—
d4601	Moving around within buildings other than home	—	—	UCSD-SBQ^	—	—	—	—	—	—	—	—	—
d4602	Moving around outside the home and other buildings	—	—	—	—	—	SGRQ	—	—	LIPF	—	—	—
d5	General self-care	—	—	—	—	—	—	—	K-BILD	LIPF	—	—	—
d510	Washing oneself	—	—	—	—	—	SGRQ	—	—	LIPF	—	—	—
d5101	Washing whole body	—	—	—	—	SF-36	—	—	—	—	—	—	—
d5109	Washing oneself, unspecified	—	—	UCSD-SBQ^	—	—	—	—	—	—	—	—	—
d520	Caring for body parts	—	—	—	—	—	—	—	—	LIPF	—	—	—
d5201	Caring for teeth	—	—	UCSD-SBQ^	—	—	—	—	—	LIPF	—	—	—
d5202	Caring for hair	—	—	UCSD-SBQ^	—	—	—	—	—	LIPF	—	—	—
d540^a^	Dressing	—	—	UCSD-SBQ^	—	SF-36	—	—	—	LIPF	—	—	—
d550	Eating	—	—	UCSD-SBQ^	—	—	—	—	—	—	—	—	—
d570^a^	Looking after one’s health	—	—	UCSD-SBQ^	—	—	SGRQ	—	—	LIPF	—	—	—
d6	Domestic Life	—	—	—	—	—	—	—	K-BILD	LIPF	—	—	—
d6200	Shopping	—	—	UCSD-SBQ^	—	—	SGRQ	—	—	—	—	—	—
d640^a^	Doing housework	—	—	UCSD-SBQ^	—	SF-36	SGRQ	—	—	—	—	—	—
d6400	Washing and drying clothes and garments	—	—	UCSD-SBQ^	—	—	—	—	—	—	—	—	—
d6402	Cleaning living area	MRC	—	UCSD-SBQ^	—	—	—	—	—	—	—	—	—
d6403	Using household appliances	—	—	UCSD-SBQ^a^	—	—	—	—	—	—	—	—	—
d6408_mowing lawn	Doing housework, other specified	—	—	UCSD-SBQ^	—	—	—	—	—	—	—	—	—
d6408_shovel snow	Doing housework, other specified	—	—	—	—	—	SGRQ	—	—	—	—	—	—
d6408_watering lawn	Doing housework, other specified	—	—	UCSD-SBQ^	—	—	—	—	—	—	—	—	—
d6503	Maintaining vehicles	—	—	UCSD-SBQ^	—	—	—	—	—	—	—	—	—
d6505	Taking care of plants, indoors and outdoors	—	—	UCSD-SBQ^	—	—	SGRQ	—	—	—	—	—	—
d7702	Sexual relationships	—	—	UCSD-SBQ^	—	—	—	—	—	—	—	—	—
d845	Acquiring, keeping and terminating a job	—	—	—	LCQ	—	—	—	—	—	—	—	—
d8451	Maintaining a job	—	—	—	LCQ	—	—	—	K-BILD	—	—	—	—
d850^a^	Remunerative employment	—	—	—	LCQ	SF-36	—	—	K-BILD	—	—	—	—
d855	Non-remunerative employment	—	—	—	LCQ	—	—	—	—	—	—	—	—
d9	Community, social and civic life	—	—	—	—	—	—	—	K-BILD	LIPF	—	—	—
d920	Recreation and leisure	—	—	—	—	—	SGRQ	—	—	—	—	—	—
d9200	Play	—	—	—	—	—	SGRQ	—	—	—	—	—	—
d9201	Sports	—	—	—	—	SF-36	SGRQ	—	—	—	—	—	—
d9202	Arts and culture	—	—	—	—	—	SGRQ	—	—	—	—	—	—
d9205	Socializing	—	—	—	—	SF-36	—	—	—	—	—	—	—
**Environmental Factors**													
e115_supplemental_oxygen	Products and technology for personal use in daily living	—	—	—	—	—	—	—	—	LIPF	—	—	—
e1650	Financial assets	—	—	—	—	—	—	—	K-BILD	—	—	—	—
e1651	Tangible assets	—	—	—	—	—	—	—	K-BILD	—	—	—	—
e2100	Land forms: Features of land forms, such as mountains, hills, valleys and plains	—	—	—	—	—	SGRQ	—	—	—	—	—	—
e2450	Day/night cycles	—	—	—	—	—	SGRQ	—	—	—	—	—	—
e260	Air quality	—	—	—	LCQ	—	—	—	—	—	—	—	—
e340	Personal care providers and personal assistants	—	—	—	—	—	—	—	—	LIPF	—	—	—
e410	Individual attitudes of immediate family members	—	—	—	LCQ	—	SGRQ	—	—	—	—	—	—
e415	Individual attitudes of extended family members	—	—	—	—	—	SGRQ	—	—	—	—	—	—
e420	Individual attitudes of friends	—	—	—	LCQ	—	SGRQ	—	—	—	—	—	—
e425	Individual attitudes of acquaintances, peers, colleagues, neighbours and community members	—	—	—	—	—	SGRQ	—	—	—	—	—	—
e460	Societal attitudes	—	—	—	LCQ	—	—	—	—	—	—	—	—

MRC: medical research council dyspnea scale; D-12: Dyspnea-12, UCSD-SBQ: University of California Shortness of Breath Questionnaire, LCQ: Leicester Cough Questionnaire, SF-36: IPF-PROM: Pulmonary Fibrosis-Patient-Reported Outcome Measure; K-BILD: King’s Brief ILD, questionnaire, LIPF: Living with IPF Questionnaire, Medical Outcomes Study Short-Form 36, VAS-PG: visual analogue scale patient global assessment of disease activity; HRCT: extent of interstitial disease on high resolution computed tomography; FVC: forced vital capacity; DLCO: diffusion capacity of lung for carbon monoxide. All instruments apply to both IPF, and CTD-ILD, unless where indicated.

a Indicates an ICF, category with linkages to 3 or more instruments. ^Indicates the consensus instrument was for IPF, only.

**TABLE 5:** Categorical concepts unique to a single instrument (*Courtesy LA Saketkoo, with permission, rights reserved.*)

ICF descriptor	ICF category	Instrument
Structure of lungs	s4301	HRCT
Psychic Stability	b1263	SF-36
Emotional Functions	b152	SF-36
Pain in stomach or abdomen	b28012	LCQ
Quality of voice	b310	LIPF
	b3101	LCQ
Respiration functions not specified: cough with deep inspiration	b4408_cough with deep inspiration	LIPF
Sensations associated with cardiovascular and respiratory functions	b460_air hunger/gasp	K-BILD
	b460_chest tightness	K-BILD
	b460_wheeze/whistling sound	K-BILD
Additional sensations of the cardiovascular/respiratory system specified	b469_at rest	LIPF
	b469_bouts of coughing	LCQ
	b469_cough/ing with deep inspiration	LIPF
	b469_cough/ing with over-exertion	LIPF
Pace Self Throughout Day	d2309_pace self	LIPF
Speaking	d330	LCQ
Using telecommunication devices	d3600	LCQ
Kneeling	d4102	SF-36
Fine hand use	d440	SF-36
Changing position between sitting and standing	d4103, d4104	UCSD-SBQ
Hand/arm use	d445	SF-36
Moving around outside the home	d4601	UCSD-SBQ
Eating	d550	UCSD-SBQ
Sexual Relationships	d7702	UCSD-SBQ

The *UCSD-SBQ* is the only included PROM to query self-nourishment (*d550*) and sexual activity (*d7702*). Concepts of financial solvency, such as maintaining remunerative employment and assets were only queried in the *K-BILD* and *LCQ*.

### Personal and environmental factors

Thirty-four “*Personal Factors*” (Table 6) reflected disease-specific qualities not described elsewhere in the ICF, e.g. “pf_embarrassed by cough” or “pf_panic/afraid when can’t get a breath”. “*Personal Factors*” mainly captured the emotional impact of living with ILD ranging from episodic feelings such as panic, fright, distress, frustration and embarrassment; to those of more goading nature such as fear, worry, agitation; and more chronic undercurrents of emotion such as anxiety, coping with uncertainty, fear of symptoms and thinking about death. “*Personal Factors*” also described perceptions of health status such as quality of life and frailty, “*Environmental Factors”* related to the attitudes of others (family *e410/e415*, friends *e420*, acquaintances *e425*, societal *e460*) potentially impacting impairment were predominantly represented by the *LCQ* and *SGRQ*. *SGRQ* uniquely queried terrain (*e2100*) and circadian timing of symptoms (*e2450*). *LCQ*, *K-BILD* uniquely queried air quality (*e260*) and assets (*e1650*, *e1651*), respectively. *LIPF* uniquely queried about products/technology for personal use (*e111_supplemental oxygen*) and personal care providers (*e340*).

**TABLE 6:** Additional considerations for ICF ILD linkages from instruments and domains (*Courtesy LA Saketkoo, with permission, rights reserved*)

ICF components	Newly proposed descriptors	Instrument
Health Condition	General Health	SF-36
	HC_chest condition	SGRQ, K-BILD
	HC_chest problem	SGRQ
	HC_IPF	LIPF
	HC_lung complaint	K-BILD
	HC_lung disease	K-BILD
	HC_pulmonary fibrosis	PF/IPF-PROM
Not Defined	Mortality_nd	MORTALITY
	VAS-PG_nd	VAS-PG
	nd_getting worse	K-BILD
	nd_how much of the time	K-BILD
	nd_a problem	LIPF
	nd_day to day life	LIPF
	nd_hassle	LIPF
	nd_need to rest	LIPF
	nd_physical activity	LIPF
	nd_tickle in throat	LIPF
Personal Factors	pf_afraid/panic when can’t get breath	SGRQ
	pf_agitated	D-12
	pf_annoying	LIPF
	pf_anxious	LCQ, K-BILD
	pf_avoid	K-BILD
	pf_cope with uncertainty	PF/IPF-PROM
	pf_cough caused worry about illness	LCQ
	pf_cough interfered with joy of life	LCQ
	pf_depressed	D-12, K-BILD
	pf_distressing	D-12
	pf_embarassing/ed	SGRQ, LIPF
	pf_embarrassed by cough	LCQ
	pf_exercise not safe	SGRQ
	pf_expected/anticipated	K-BILD
	pf_fear	LIPF
	pf_fear of hurting self by overexertion	UCSD-SBQ*
	pf_fear of shortness of breath	UCSD-SBQ*, PF/IPF-PROM
	pf_fed up	LCQ, K-BILD
	pf_felt in control	K-BILD
	pf_feel in control of cough	LCQ
	pf_frail/invalid	SGRQ
	pf_frightening	LIPF
	pf_frustrated	PF/IPF-PROM, LIPF
	pf_frustrated by cough	LCQ
	pf_frustrated by being tired	PF/IPF-PROM
	pf_get sick easier than others	SF-36
	pf_irritating	D-12, K-BILD
	pf_miserable	D-12
	pf_not in control of chest problem	SGRQ, K-BILD
	pf_quality of life	LIPF
	pf_think about death	K-BILD
	pf_worry	PF/IPF-PROM
	pf_worried about serious illness	K-BILD

## Discussion

Herein, we provide a reference of 111 ICF categories describing impairment in ILD for use in the clinical setting with potential transferability for clinical trial use, especially with regard to optimization of PROMs. The importance of ICF Core Sets is heightened in rare or commonly misunderstood diseases and their manifestations, as they are intended to provide an assembly of biophysical and psychosocial features relevant and important to a health condition. In so doing, they can provide a clinical focus for patient experiences of disease that may often go under-recognized; and can potentially be teaching tools to familiarize clinicians with the patient experience of rarer diseases.

Disability is the impact of a health condition on a person’s global functioning characterized by body-level impairments, society-level participation limitations and impact on psychological well-being. “*Activities and Participation*”, representing >70% of the linkages identified in our study, is possibly the most relevant ICF component to a patient’s experience of disability. This was demonstrated in previous studies (Saketkoo et al., 2014a; Saketkoo et al., 2014b) where patients with ILD almost exclusively discuss their condition in terms of activity and life participation aspects.

Considering real-world examples of an ICF Core Set or Checklist can help illustrate utility. An initial evaluation of a person with ILD in pulmonary rehabilitation, for example, elicits the top three concerns of living with ILD of which “*coughing spells*” is stated by the patient as the most pressing priority. The therapist advises that there are several strategies that can be taught and practiced in pulmonary rehabilitation to help manage and recover from coughing spells. The therapist might then work with the patient using the Core Set in ILD to identify the relevant categories that reflect life activities that they feel are most impaired by their experience of cough. Each of these patient-indicated categories can then be monitored over time via use of a numeric rating scale (NRS). The presentation of the Core Set helps support patient discussion in developing a personalized tool to gauge patient-perceived progress in areas that are of high priority for the patient. During the therapist’s query the patient expresses *urinary incontinence* during coughing spells has become a major issue. Though *urinary incontinence* may not be a part of the Core Set for ILD, it will be added to the patient’s list as a complication of cough that will be monitored over time.

The ICF linkage process, however, is limited in its transferability. For example in the case of PROM, ICF linkage demands that each question from a PROM be dissected into its single-concept units, with each linkage reflecting one concept of a question-item. Although inferences from an entire question-item can be made, the process does not accommodate joining of concept units to reflect the entirety of a question-item’s meaning.

ICF categories are dual-edged in that they are specific yet also generic. Each ICF category is sufficiently generic and able to be linked to many different health conditions (e.g. “*ability to concentrate*”, *b140*, may apply to diabetes, heart failure, traumatic brain injury, etc.), thus providing a comprehensive yet feasible system for tracking a type of disability. Whereas, the specific nature of the categories has potential to accurately capture the nature of symptomatic impairment. For example, *b4402*, “*depth of inspiration”* is a highly granular descriptor of a respiratory function (*b440*), and further still the following categories describing respiratory muscle function (*b445*) alone or in combination distinctively characterize respiratory muscle weakness: b4450, “*thoracic”* versus *b4451*, “*diaphragmatic”* versus *b4452*, “*accessory*” respiratory muscle function.

Some areas of ICF categories, can also be abstract, and devoid of contextual life circumstances. Such bare generic descriptions confer feasibility for clinical documentation purposes but pose limitations on accuracy and meaningfulness of patient query. In contrast, a single question from a PROM often contains several converging concepts reflective of a patient’s experience of that impairment, and thus an isolated ICF category may not hold strong patient-reported relevance a potential vulnerability in the ICD-11 implementation. The ICF is an evolving system that can tolerate expansion to include ICF categories that more closely reflect patient experiences of specific impairments and, thus, improve accuracy of patient responses.

Most included PROMs were developed with careful qualitative methods but have yielded significant conceptual variation. For example, the *Dyspnea-12 (D-12)* and *L-IPF* provide an ICF category, *b4402*, “*depth of respiration”*, which is a ubiquitous concern of patients with ILD (Saketkoo et al., 2014a; Saketkoo et al., 2014b) yet appears only in these two PROMs. This is also true of *d7702*, “*sexual relationships”*, which is meaningful to patients but only found in the *USCD-SBQ*. This lack of capture occurs in other health conditions, such as myositis, where *intimacy* and *sexual relations* are of high patient-reported importance in more private data-collection circumstances, such as surveys or semi-structured interviews. Patients may be more reticent to supply ultra-sensitive information in larger focus groups (Alexanderson et al., 2002; Alemo Munters et al., 2011).

In contrast, single-occurring linkages may also demonstrate weak relevance to the specific disease. In this study, *d440*, “*fine hand use”* from the *SF-36*, does not provide information relevant to ILD. However, the reason for continued use of a generic measure like the *SF-36* lies in its global validation across diseases, making it an essential anchor and comparator.

The utility of an ICF Core Set in ILD also enables application to a CTD-ILD. Using the example of RA for which an ICF Core Set already exists, the current recommendation for RA-ILD would be to combine two separate ICF Core Sets, one for ILD and the RA (Stucki and Cieza, 2004). The development of ICF Core Sets in more multi-organ system predominant conditions like systemic sclerosis (SSc), idiopathic inflammatory myopathies (IIMs) or sarcoidosis, are likely to incorporate an ILD ICF Core Set into their frameworks (Saketkoo et al., 2012b; Saketkoo et al., 2012c).

An important example highlighting the influence of analytic approach on patient-relevant concepts arose during *K-BILD* development. Academic curiosity spurred applications to the original dataset using Rasch analysis (resulting in the currently circulating *K-BILD-R*) and also item response theory resulting in *K-BILD-I*. Remarkably, only *K-BILD-R* retained the questions regarding financial solvency; while only *K-BILD-I* retained items on fatigue, a predominant concept in other ILD PROMs and both are crucial concepts for people living with ILD. This finding led to group discussion regarding “fatigue” persistently being relegated to a function of HRQoL in ILD. While it was agreed that there is value in re-examining “fatigue” in ILD as a complex and multi-dimensional core symptom domain (De Vries et al., 2000; De Vries et al., 2001; De Vries and Drent, 2006; Saketkoo et al., 2014a; Hendriks et al., 2018; Kølner-Augustson et al., 2020).

The ICF linkage provides an expansive view of the great wealth of these PROMs that may together be harmonized into a streamlined instrument incorporating the optimal aspects of each. Such an effort would entail wide global engagement of patients and patient partners in tightly iterative applications of consensus methodology and testing (Stucki and Cieza, 2004).

### Recommendations for lung disease in the WHO ICF classification system

The ICF was designed to be broadly comprehensive rather than detail exhaustive. The ICF structure was intended to be responsive to modification and development over time, exemplified by the ICF evolution of “*Personal Factors”*. Our instrument linkage demonstrated essential symptoms related to chronic pulmonary and cardiovascular diseases that require consideration for inclusion in future ICF updates. Three of these essential areas are described below.

#### Biophysiologic mechanisms discussion

Cough is an intrinsic experience creating significant impairment in pulmonary disease. In ILD a restrictive physiology, inspiratory and predominantly dry cough creates significant interference in life activities. Phlegm production, though not as frequent or troubling in ILD as in COPD, is an essential descriptor of cough. Temporary placeholder categories were created through this investigation (e.g. b450_xx, b460_ xx or b469_xx) to capture the intrinsic in ILD and lung disease that currently lack sufficient representation in ICF language. Enduring additions to ICF were proposed to directly designate cough, inspiratory cough, dyspnea, wheeze, air hunger and phlegm production.

#### Psychosocial function discussion

Though not a direct bio-physical manifestation of pulmonary disease, patients perceive psychosocial impact as an intrinsic experience of ILD (De Vries et al., 2000; Swigris et al., 2005; Saketkoo et al., 2014a; Saketkoo et al., 2014b), especially when biophysical symptoms are present with rest or slight exertion, resulting in disabling breathlessness or cough and which appear to be deeply entwined with embarrassment, frustration, fear, safety with exertion, and loss of control. Capturing and describing the disease-related psychological impact of a non-psychiatric health condition is a current challenge within the ICF.

Several instruments contain question-items that measure degrees of frustration, disgust, embarrassment, distress, fear or sense of safety with exertion. Embarrassment, one such frequent descriptor, as an example, greatly impacts psychological functioning and coping with cough which is a ubiquitous experience drawing both visual and auditory unwanted attention in ILDs (De Vries et al., 2000; Swigris et al., 2005; Key et al., 2010; Jones et al., 2011; Theodore et al., 2012; Saketkoo et al., 2014a; Saketkoo et al., 2014b) and other lung conditions. These concepts are strongly echoed in ILD patient qualitative data (De Vries et al., 2000; Swigris et al., 2005; Saketkoo et al., 2014a; Saketkoo et al., 2014b) and perceived to influence level of functioning. These would be important dynamics for an ILD ICF Core Set but are yet awaiting evolution within ICF under the “personal factors” component.

#### Physical function discussion

The ICF’s generic nature can impede accurate symptom query and may warrant added contextualization of ICF descriptors. Activity-descriptors common in respiratory-related PROMs and patient-reported visit history that require significant cardiopulmonary exertion, such as mowing lawn and shoveling snow, are not sufficiently defined by current ICF categories. The closest combined categories for “mowing lawn” are d4551 “pushing” which is intended to describe upper extremity function buts lacks sufficient discrimination between cardiopulmonary, muscular or joint capability and d6505 “taking care of outdoor plants’. Elements of cardiopulmonary recovery (stopping, resting mid-activity), pace (e.g. performing more slowly; potentially related to recovery); punctuation of disease behavior (such as “attack”, “flaring”) as well as frequency of symptoms are temporal and dynamic associations essential to pulmonary disease requiring future ICF updates.

A proposal for future ICF revisions, is the addition of a discrete category or an additional component to the ICF that describes the global perception of disease burden from the patient’s perspective. The *VAS-PtG* is widely validated across many diseases as a reliable marker, sensitive to change and correlative with objective measures of disease activity (Singh et al., 2011; Bartlett et al., 2012). Its inclusion into the ICF could enhance report of perceived function and burden of disease as well as the incremental impact of modification of environmental factors.

### Future steps

This study’s identification of 111 ICF categories, 34 “*personal factors”* and multiple further descriptors under “*health condition”* (*hc*) and “*not defined*” (*nd*) will develop into a manageable ICF Core Set over time (Kostanjsek et al., 2011; Finger et al., 2014). Our continuance of iterative multi-disciplinary methodological applications that include patient research partners as essential team members will refine and identify the most relevant and important concepts of both the somatic and psychosocial realms experienced by people living with ILD. Pursuant to this is working with the WHO ICF to address the expansion of descriptors to accurately reflect functional impairment intrinsic to living with ILD. Discussions amongst the authors presented creating central pathways to assess side effects of disease-related treatment beyond being under “*environmental factors”* but rather under “b” and “d” categories (Proesmans et al., 2019). Finally, the dedicated efforts in the development of past PROMs have provided a wealth of information that can result in a potentially streamlined exquisitely responsive instrument.

## Conclusion

This is the first effort to examine ICF Core Sets in ILD. This investigation provided an important and useful step to facilitating clinician preparation for ICD-11 and other performance quality assessments that will require ICF use (Iezzoni and Greenberg, 2003; Health and Human Services/Centers for Medicare and Medicaid Services, 2000; SocialSecurity Administration, 2013; Escorpizo and Stucki, 2013). The utility of disease-specific ICF Core Sets is multi-factorial on individual, regional and global levels offering value to epidemiologic, health economics, clinical assessment, PROM development and comparison for fair representation in policy, service provision and research funding assessments as well as the potential development of concise PROMs. Future steps may build on harmonizing these PROMs to widely validate concepts, context and language in ILD. Our investigation identifies new ICF categories, for general pulmonary disease to be considered in the future ICF revisions.

## Data Availability

The original contributions presented in the study are included in the article/Supplementary Material. Further inquiries can be directed to the corresponding authors.

## Abbreviations

CM, Center for Medicare and Medicaid Services, CTD, Connective Tissue Disease; DLCO, Diffusion Capacity of Lung for Carbon Monoxide; FVC, Forced Vital Capacity; HRCT, High Resolution Computed Tomography; ICD, International Classification of Diseases; ICF, International Classification of Functioning Disability and Health; IIP, Interstitial Idiopathic Pneumonitis; IPF, Idiopathic Pulmonary Fibrosis; PF/IPF-PROM, Pulmonary Fibrosis/Idiopathic Pulmonary Fibrosis Patient Reported Outcome Measure; ILD, Interstitial Lung Disease; K-BILD, King’s Brief Interstitial Lung Disease Questionnaire; L-IPF, Living with IPF; PSFS, Patient Specific Functional Scale; PROM, Patient-Reported Outcome Measures; PF, Pulmonary Fibrosis; pf, Personal factors; SF-36, Short Form 36; UCSD-SBQ, University of California-Shortness of Breath Questionnaire; WHO, World Health Organization.
