# Supplementary material for: World Health Organization (WHO) International Classification of Functioning, Disability and Health (ICF) Core Set Development for Interstitial Lung Disease
Source: Front Pharmacol. 2022 Oct 14;13:979788. doi: 10.3389/fphar.2022.979788 (PMC9615472; doi:10.3389/fphar.2022.979788)
Supplement: Supplementary file 1 [file DataSheet1.docx]

**ONLINE SUPPLEMENT**

Lesley Ann Saketkoo, MD, MPH^1,2,3,4^, Reuben Escorpizo, PT, DPT, MSc^5,6^, Janos Varga, MD, PhD^7^, Kevin J. Keen, PhD, PStat^8,9^, Kim Fligelstone^10,11^, Surinder S. Birring, MD^12^, Helene Alexanderson, PhD^13,14^; Henrik Petterson, PhD^13,14^; Humza Ahmad Chaudhry^1,2,3^, Janet Poole, PhD^15^; Malin Regardt, PhD; ^13,14^ Daphne LeSage^16^, Catherine Sarver^17^; Joseph Lanario, MSc, BSc^18^; Elisabetta Renzoni, MD^19^, Mary Beth Scholand, MD^20^, Matthew R. Lammi, MD^1,2,4^, Otylia Kowal-Bielecka, MD^21^, Oliver Distler, MD^22^, Tracy Frech, MD, MPH^23,24^; Lee Shapiro, MD^25,26^; Cecilia Varju’,MD, PhD^27^; Elizabeth R. Volkmann, MD^28^; Elana J. Bernstein, MD, MS^29^; Marjolein Drent, MD, PhD^30,31^; Ogugua Ndili Obi, MD, MPH^32^; Karen Patterson, MD^33,34^; Anne-Marie Russell, RN, PhD^35,36^; *In collaboration with The Global Fellowship on Rehabilitation and Exercise in Systemic Sclerosis (G-FoRSS)*

^1^New Orleans Scleroderma and Sarcoidosis Patient Care and Research Center;

^2^University Medical Center – Comprehensive Pulmonary Hypertension Center & Interstitial Lung Disease Clinic Programs, New Orleans, LA, USA

^3^Tulane University School of Medicine, New Orleans, LA, USA

^4^Louisiana State University Health Sciences Center, Division of Pulmonary Medicine – New Orleans, LA, USA;

^5^Department of Rehabilitation and Movement Science, The University of Vermont, Burlington, VT, USA;

^6^Swiss Paraplegic Research, Nottwil, Switzerland;

^7^Department of Pulmonology, Semmelweis University, Budapest, Hungary

^8^Department of Mathematics and Statistics & the ^9^Health Research Institute University of Northern British Columbia, Prince George, Canada;

^9^Department of Medicine, University of British Columbia & Centre for Heart Lung Innovation, Providence Research, Vancouver, Canada;

^10^Patient Research Partner Scleroderma & Raynaud Society, UK (SRUK) and Federation of European Scleroderma Associations, UK

^11^Royal Free Hospital Scleroderma Unit, London, UK;

^12^Division of Asthma, Allergy and Lung Biology, King’s College London, London, United Kingdom,

^13^Women’s Health and Allied Health Professionals, Medical Unit Occupational Therapy and Physiotherapy, Karolinska University Hospital, Stockholm, Sweden

^14^Division of Rheumatology, Department of Medicin, Solna, Karolinska Institutet, Stockholm, Sweden

^15^Occupational Therapy Graduate Program, University of New Mexico, Albuquerque, NM, USA

^16^Patient Research Partner, New Orleans, USA;

^17^Patient Research Partner, Baltimore, MD, USA

^18^Research Fellow in Respiratory Health – Exeter Respiratory Institute Royal Devon University Hospitals NHS Foundation Trust, Exeter, UK

^19^Royal Brompton Hospital, National Heart and Lung Institute, London, UK;

^20^Pulmonary Medicine, University of Utah, Salt Lake City, United States,

^21^University of Bialystok, Bialystok, Poland;

^22^Division of Rheumatology, University Hospital Zurich, Switzerland;

^23^Division of Rheumatology Vanderbilt University School of Medicine, Nashville, TN; USA

^24^Pulmonary Medicine, University of Utah, Salt Lake City, United States,

^25^Division of Rheumatology, Albany Medical Center, Albany, NY, USA

^26^Steffens Scleroderma Foundation, Albany, NY, USA

^27^Department of Rheumatology and Immunology, University of Pécs Clinical Center, Pecs, Hungary

^28^University of California, David Geffen School of Medicine, UCLA Scleroderma Program and UCLA CTD-ILD Program, Division of Rheumatology, Department of Medicine, Los Angeles, CA, USA

^29^Columbia University/New York-Presbyterian Scleroderma Program, Division of Rheumatology,

Department of Medicine, Columbia University College of Physicians and Surgeons, New York, NY, USA

^30^Interstitial Lung Diseases (ILD) Center of Excellence, Department of Pulmonology, St. Antonius Hospital, Koekoekslaan 1, 3435 CM Nieuwegein, The Netherlands.

^31^Department of Pharmacology and Toxicology, Faculty of Health and Life Sciences, Maastricht University, 40, 6229 ER Maastricht, The Netherlands.

^32^Division of Pulmonary, Critical Care and Sleep Medicine, Department of Internal Medicine, Brody School of Medicine, East Carolina University, Greenville, North Carolina, USA.

^33^Brighton & Sussex Medical School, Department of Clinical & Experimental Medicine, Falmer, UK

^34^Division Pulmonary, Allergy, and Critical care medicine, Perelman School of Medicine, University of Pennsylvania, Philadelphia, Pennsylvania

^35^Respiratory Institute, University of Exeter, Exeter, UK

^36^Respiratory Medicine, Royal Devon University Healthcare NHS Foundation Trust. London, UK

**Correspondence**: Anne-Marie Russell, PhD, ARNP

Exeter University

Exeter, UK

Email: a.russell4[at]exeter.ac.uk

Lesley Ann Saketkoo, MD, MPH

New Orleans Scleroderma and Sarcoidosis Patient Care and Research Center

2001 Canal Street; New Orleans, Louisiana 70112, USA

Email: lsaketk[at]tulane.edu

**ICF Classification System**

*History*

The need for the scientific collection of disability data was recognized after the advent of the WHO’s Global Burden of Disease Initiative (GBD) in 1990. The WHO-GBD introduced the 9^th^ revision of the International Classification of Diseases (ICD-9). The ICD-9/GBD framework illuminated geographical clustering of disease and infrastructure challenges to achieving improved health outcomes. The WHO-GBD developed the disability-adjusted-life-year (DALY) metric and generated data using the ICD-9 and the DALY that powered targeted global public health interventions. This resulted in remarkable changes in disease incidence and death related to preventable—mostly infectious—diseases. Since then, the ICD has grown beyond its intended use. It is now almost exclusively known as a diagnostic coding system for reimbursement with which virtually every practicing clinician engages.

The positive impact of the public health interventions prompted by the WHO-GBD resulted in recognition of a global signal in *chronic* illness and disability–most of which was due to traumatic injury and cardio/cerebrovascular disease–and presumed to be impacting global economic productivity. In order to assess and understand the impact related to this newly-recognized prevalence of chronic disease, the WHO developed a framework, the International Classification of Functioning, Disability and Health (ICF) to describe and quantify impairment with a standardized scientific method of disability data collection. The goal of which was to inform healthcare policies, guide provision of health services and overcome urban and rural infrastructure barriers to health services that optimize engagement in important life areas including remunerative employment.

**The ICF**

The ICF is a hierarchy of over 1200 ‘categories’ or codes belonging to discrete components used to depict functioning and disability of an individual. There are two over-arching parts: *Functioning and Disability*, and *Contextual Factors*. *Functioning and Disability* is the predominant operational part of the ICF and consists of ‘*body function’*, ‘*body structure’*, and ‘*activities and participation’*. *Contextual Factors* are divided into external or ‘*Environmental Factors’* and internal or ‘*Personal Factors*’ (Fig. 1).

**Figure 1. Interactions between domains of ICF in relation to the health condition.** *(Courtesy of LA Saketkoo, permission granted, rights reserved)*


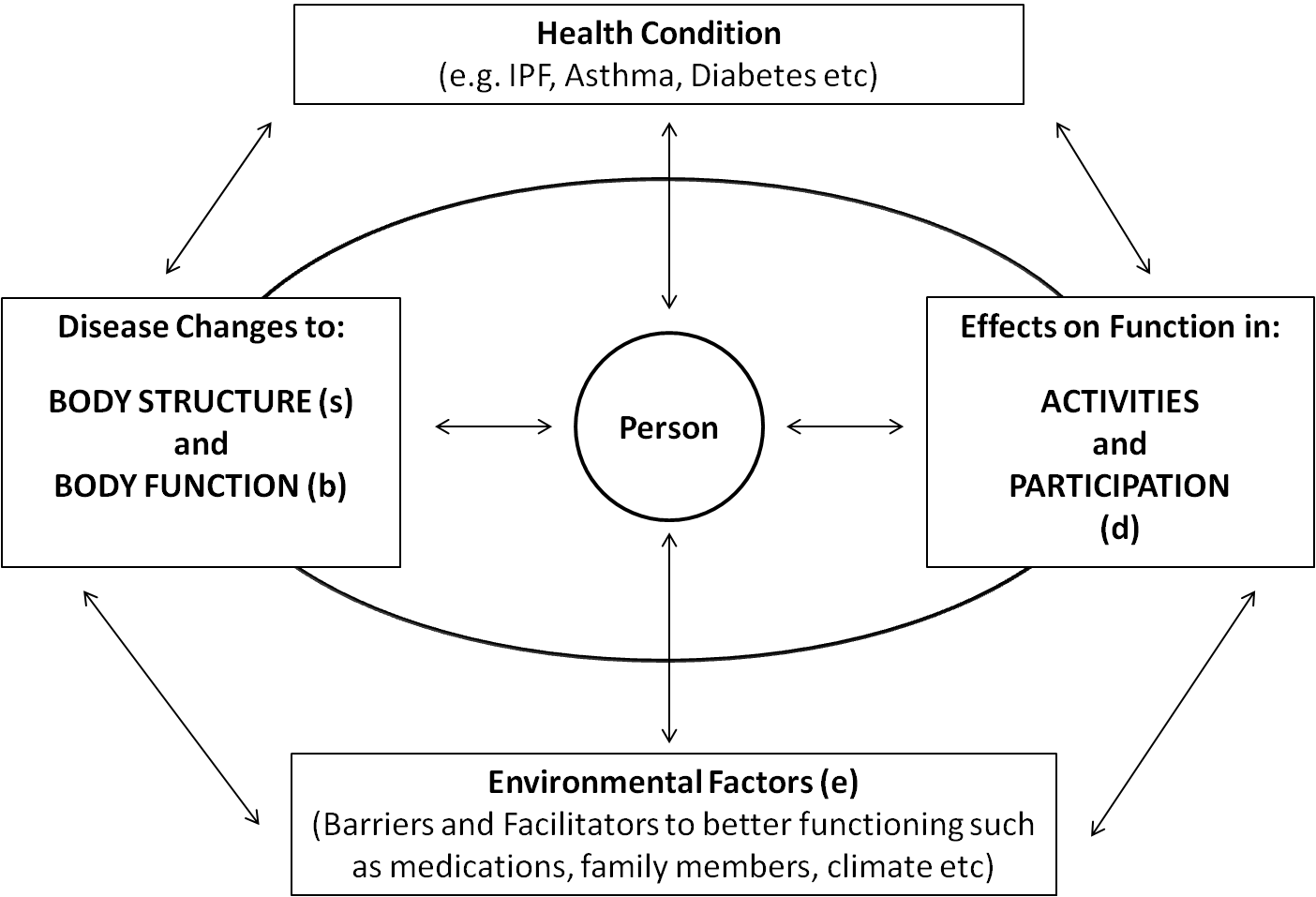


**Functioning and Disability**

‘*Body structure*’ - described under the ‘**s**’ terms, relate to *abnormalities of anatomical structure* whether gross, histological or radiological assessment, such as abnormalities of lung parenchyma (s4301) or in finger joints (s73021).

‘*Body function*’ - described under the ‘**b**’ terms, and defined as physiologic functions of the body systems which includes *symptomatic experience* of physical, mental and emotional functions such in what aspect the function of the heart, gastrointestinal or respiratory system does not work e.g. dyspnea (b460), cough (b450) or chest pain (b28011).

‘*Activities and participation*’ organized under the ‘**d**’ terms, with ‘activities’ defined as execution of task or action by an individual and involvement in a life situation such as lifting (d4300), dressing (d540), bathing (d510), cooking (d630), bending (d4105) or moving between locations (d460) and ‘participation’ defined as involvement in life situations such as work (d850), college (d830), family (d760) and social life (d750). The performance of activities and participation is the interface where abnormalities in structure/function precipitate as a disability – when a person attempts to interact with the many activities of life. An example of this would be, showering impaired by severe dyspnea or buttoning impaired by symptoms of rheumatoid arthritis.

**Contextual Factors**

‘*Environmental factors*’ (external factors) - under ‘**e**’ terms - describe factors that influence one’s ability. The same codes apply to both facilitators, e.g. attitudes of immediate family members (e410^+^), medications or oxygen (e1101^+^), and assistive devices (e1151^+^); or barriers e.g. stressful family relations (e410), social attitudes (e460), stairs (e150), lack of income (e1650), cold climate (e2250), distance from health services (e5800). Facilitators are differentiated by a ‘+’ in the notation.

Each of these domains deconstructs disability into chapters which are the 1^st^ level of classification under which impairment, limitation, or restriction can be hierarchically detailed in further levels of classification. For example, ‘dysphagia’ would be: ‘b51052’; whereby:

‘b5’ is the body functions chapter ‘*Functions of the digestive, metabolic and endocrine systems*’,

‘b510’ is the 2nd level classification of ‘*Ingestion functions’*,

‘b5105’ is the 3rd level of ‘*Swallowing’*; and

‘b51052’ is the detailed descriptor of ‘*Esophageal swallowing’*.

The degree of disability may be reported using the ICF prescribed rating scale (no, mild, moderate, severe and complete disability).

‘*Personal factors’* (internal factors) are a developing area of the ICF and are individualized factors independent of the health condition that may have an influence on disability–the impact of which is presumed to vary depending on location and strength of culture. These factors may include gender, race, age, co-morbidities, lifestyle, coping styles, social background, behavior and psychological characteristics.
